# Disseminated Gonococcal Infection: An Unusual Presentation Resembling a Lupus Flare

**DOI:** 10.7759/cureus.28471

**Published:** 2022-08-27

**Authors:** Emily Barton, Nicole Melendez

**Affiliations:** 1 Osteopathic Medicine, Lake Erie College of Osteopathic Medicine (LECOM) Bradenton, Bradenton, USA; 2 Rheumatology, St. Joseph's Hospital, Tampa, USA

**Keywords:** polyarthritis, systemic lupus erythematosus, disseminated gonococcal infection, case study, n. gonorrhoeae

## Abstract

We present a misdiagnosed case of disseminated gonococcal infection (DGI) in a patient with pre-existing systemic lupus erythematosus (SLE), complaining of sudden onset polyarthralgia and tenosynovitis. *Neisseria gonorrhoeae* is a common sexually transmitted disease. It can present in young adults as cervicitis and urethritis but is often asymptomatic. In rare instances, gonorrhea can advance to DGI with symptoms such as tenosynovitis, dermatitis, and polyarthralgias, seen most commonly in the knees, ankles, elbows, fingers, and toes. Once suspected, DGI is definitively diagnosed with blood cultures or synovial fluid analysis. SLE is associated with an increased risk of disseminated infections, including DGI. Therefore, early diagnosis of DGI is critical for successful treatment and recovery. Providers should therefore be conscientious of the overlap in symptoms between DGI and a lupus flare. The purpose of examining this case is to encourage the inclusion of disseminated *N. gonorrhoeae* infection as a differential diagnosis in SLE patients presenting with acute arthralgias regardless of genitourinary symptoms.

## Introduction

*Neisseria gonorrhoeae* is a gram-negative diplococcus that causes gonorrhea and is transmitted during vaginal, oral, and anal intercourse. Localized manifestations of the disease include vaginal discharge and changes in urinary frequency or pain. Most patients with uncomplicated gonorrhea respond well to a 500 mg ceftriaxone intramuscular injection [1]. However, in 0.5-3.0% of cases, patients will develop disseminated gonococcal infection (DGI) that can progress to septic arthritis in 42%-85% of patients [2,3]. Complement deficiencies and systemic lupus erythematosus (SLE) coincide and are some of the factors known to increase the risk of gonorrhea and DGI [3]. In this case study, the patient initially presented with symptoms consistent with a lupus flare that was later revealed to be DGI.

## Case presentation

We present a case of a 20-year-old African American female with a seven-year history of SLE and biopsy-proven class IV and V lupus nephritis, who presented to the ED with a second left metatarsophalangeal (MTP) joint pain of one-day duration. Lupus disease activity was under control with belimumab, prednisone, azathioprine and hydroxychloroquine. Additional medications included nifedipine for hypertension. The patient denied any antecedent trauma and described the pain as constant and aggravated with movement. Physical exam revealed mild tenderness in the left first MTP joint. Imaging of the foot demonstrated no fractures, dislocations, or abnormalities. She was prescribed naproxen for pain and was discharged. Four days later, she returned to the ED with constant aching and burning pain on the left foot, left hand, and right knee. However, the patient's vitals remained stable. A diagnosis of gout was made, and she was prescribed colchicine, but there was no improvement. Two days later, the patient returned to the ED with worsening pain involving more joints, including both hands, the left great toe, and the right leg. No fever, night sweats, or genitourinary symptoms were reported. Physical exam showed mild swelling and tenderness around both first MTP joints, bilateral Achilles tendons, and swelling of index fingers. There was no warmth or erythema. Additional history revealed that the patient was being tapered off steroids around the time the joint pain started. A complete blood count and complete metabolic panel revealed leukocytosis, anemia, hyperkalemia, hyperchloremia, low anion gap, and low creatinine clearance. The patient was admitted with diagnoses of polymyalgia, polyarthralgia, and anemia. She was discharged two days later with the diagnosis of a lupus flare. She was instructed to continue the Medrol Dosepak, resume her home medications, and follow up with pain management and nephrology. In addition to the Medrol Dosepak, she was prescribed Norco, Voltaren topical gel, and indomethacin for acute pain.

The patient was admitted to a different hospital one week later for the fourth encounter with continued joint swelling and pain. The patient was hypertensive at 148/94, tachycardic at 118 bpm, and afebrile with an EKG showing sinus tachycardia but otherwise unremarkable. Her initial blood work showed leukocytosis, anemia, a high CRP of 4.46, and an elevated erythrocyte sedimentation rate (ESR) of 118 (Table 1). Urinalysis values were all within normal limits except for an elevated urine protein level of 20 mg/dL (Table 2). Serum complement values showed a decreased C3 of 82, positive anti-dsDNA antibodies, and positive antinuclear antibodies (ANA) (Table 3). Previous complement levels one year prior to her current symptoms showed a low C3 of 37, a low C4 of 7, and positive anti-dsDNA antibodies as high as 83.7. Her blood cultures were negative. All X-rays of her joints showed no fractures but did reveal osteopenia and possible signs of inflammatory arthropathy. The rest of the workup came back unremarkable except for a positive Aptima gonococcal DNA endocervical swab (Table 4). The asymmetric polyarthralgia and tenosynovitis on physical exam with a positive gonorrhea test led to the final diagnosis of DGI. Following this diagnosis, the patient was advised to stop azathioprine and lower the prednisone dosage to 20 mg. The DGI was treated with ceftriaxone 1 gram every 24 hours and doxycycline 100 mg twice daily. This treatment showed improvement in the joint pain and was followed by seven more days of IV ceftriaxone and oral doxycycline. Blood work at the time of discharge showed continued leukocytosis with high neutrophils and anemia (Table 1) despite improvement in symptoms. The cause of her anemia was not determined. She was discharged after seven days and followed up with rheumatology with minimal residual joint pain. 

**Table 1:** Laboratory findings of the patient. MCV: Mean corpuscular volume; MCH: Mean corpuscular hemoglobin; ESR: Erythrocyte sedimentation rate; CRP: C-reactive protein; LDH: Lactate dehydrogenase; CPK: Creatine phosphokinase; TIBC: Total iron-binding capacity; GFR: Glomerular filtration rate; BUN: Blood urea nitrogen; MCHC: Mean corpuscular hemoglobin concentration.

Component/Test	Patient Values Day 1	Patient Values Day 2	Patient Values Day 3	Patient Values Day 4	Patient Values Day 5	Patient Values Day 6	Patient Values Day 7	Reference Range
WBC	18.27 (H)	14.43 (H)	12.94 (H)	13.34 (H)	11.18 (H)	12.17 (H)	13.72 (H)	4.6-10.2 10*3/uL
RBC	3.75 (L)	3.24 (L)	3.34 (L)	3.22 (L)	3.83 (L)	3.90 (L)	3.78 (L)	4.04-5.48 10*6/uL
Hemoglobin	9.5 (L)	8.2 (L)	8.4 (L)	8.1 (L)	9.6 (L)	9.9 (L)	9.7 (L)	12.2-16.2 g/dL
Hematocrit	30.4 (L)	26.3 (L)	26.9 (L)	25.9 (L)	30.4 (L)	31.1 (L)	29.9 (L)	37-37%
MCV	81.3	81.2	80.5	80.4	79.4 (L)	79.7 (L)	79.1 (L)	80.0-97.0 fl
MCH	25.4 (L)	25.3 (L)	25.1 (L)	25.2 (L)	25.1 (L)	24.4 (L)	25.7 (L)	27.0-31.2 pg
MCHC	31.3 (L)	31.2 (L)	31.2 (L)	31.3 (L)	31.6 (L)	31.8 (L)	32.4	32-35 g/dL
Platelet Count	471 (H)	441 (H)	449 (H)	588 (H)	608 (H)	626 (H)	613 (H)	142-424 10*3/uL
MPV	10.0	11.3	10.1	10.9	10.4	10.8	10.6	9.4-12.4 fl
RDW	16.4 (H)	16.6 (H)	16.5 (H)	16.9 (H)	17.1 (H)	17.1 (H)	17.2 (H)	11.6-14.6%
% Neutrophils	74.6	76.4	61.2	61	70.9	81.0 (H)	56	39-77%
% Lymphocytes	17.7	17.4	29.9	33	21.3	13.9	33	15-47%
% Monocytes	6.5	5.7	8.1	6	5.5	3.9	8	3-13%
% Eosinophils	0.1	0.0	0.2	0	1.2	0.2	1	0-6%
% Atypical Lymphs	–	–	–	0	–	–	2 (H)	0%
Total Neutrophils	13.64 (H)	11.03 (H)	7.93 (H)	8.14	7.94 (H)	9.85 (H)	7.81	1.79-7.85 10*3/uL
Total Lymphocytes	3.24	2.52	3.87	4.40	2.38	1.69	4.46	0.69-4.79 10*3/uL
Total Monocytes	1.19	0.82	1.05	0.80	0.61	0.47	1.08	0.14-1.33 10*3/uL
Total Eosinophils	0.01	0.00	0.02	–	0.13	0.03	0.12	0.00-0.61 10*3/uL
Total Basophils	0.01	0.01	0.02	–	0.04	0.04	–	0.00-0.20 10*3/uL
Total Atypical Lymphs	–	–	–	–	–	–	0.25 (H)	0.00 10*3/uL
Sodium	136	137	142	141	–	–	136	135-148 MeQ/L
Potassium	3.9	4.0	4.0	4.0	–	–	4.0	3.5-5. 3 MeQ/L
Chloride	108 (H)	107	110 (H)	109 (H)	–	–	104	98-107 MeQ/L
CO2	20 (L)	23	24	24	–	–	24	22-29 MeQ/L
BUN	15	13	17	20	–	–	18	6-20 mg/dL
Glucose	96	88	109	100	–	–	80	70-110 mg/dL
Creatinine	0.9	0.8	0.9	0.9	–	–	0.8	0.57-1.11 mg/dL
Calcium	9.2	8.8	8.8	9.0	–	–	9.1	8.5-10.5 mg/dL
Albumin	2.2 (L)	–	–	–	–	–	–	3.5-5.9 gm/DL
Anion Gap	8	7	8	8	–	–	8	5-13 MeQ/L
BUN/Creatinine Ratio	17	16	19	22	–	–	23	–
Est GFR Non-AA	>60	>60	>60	>60	>60	>60	>60	–
Est GFR AA	>60	>60	>60	>60	>60	>60	>60	–
ESR	118 (H)	–	–	–	–	–	–	0-20 MM/HR
CRP	4.46 (H)	–	–	–	–	–	–	0.01-0.5 mg/DL
LDH	156	–	–	–	–	–	–	125-229 U/L
Reticulated Hemoglobin	28 (L)	–	–	–	–	–	–	30-38 pg
CPK	23 (L)	–	–	–	–	–	–	29-168 U/L
Iron	17 (L)	–	–	–	–	–	–	50-170 mcg/dL
TIBC	185 (L)	–	–	–	–	–	–	245-400 mcg/dL
Total Iron % Saturation	9.2 (L)	–	–	–	–	–	–	20-40%
Ferritin	61.8	–	–	–	–	–	–	4.6-204 ng/mL
Uric Acid	3.4	–	–	–	–	–	–	1-6 mg/dL
Haptoglobin	467	–	–	–	–	–	–	35-250 mg/dL
Vitamin B12	528	–	–	–	–	–	–	189-883 pg/mL
Folate	9.3	–	–	–	–	–	–	7-31.4 ng/mL

**Table 2:** Urinalysis values of the patient.

Urine Component	Patient Values Day 1	Reference Range
Glucose	Negative	Negative mg/dL
Protein	20 (Positive)	Negative mg/dL
Urobilinogen	<2	<2 mg/dL
Bilirubin	Negative	Negative mg/dL
pH	6.5	–
Ketones	Negative	Negative mg/dL
Hemoglobin	Negative	Negative
Nitrites	Negative	Negative
Leukocytes	Negative	Negative mg/dL
SG	1.006 (L)	1.007-1.030
Appearance	Clear	Clear
Color	Colorless	Yellow
RBCs	0.2	0-2/HPF
WBCs	0.5	0-5/HPF
Squamous Ep Cell	0.5	0-5/HPF
Creatinine	37.8	mg/dL

**Table 3:** Serum antibody values of the patient.

Serum Antibody	Patient Values on Admission (Day 1)	Reference Ranges
Complement C4	17.0	15-57 mg/dL
Complement C3	82 (L)	83-193 mg/dL
Hepatitis C AB	Nonreactive	Nonreactive
Hepatitis B Core Total	Nonreactive	Nonreactive
HIV Antigen AB Combo	No HIV Antigen or Antibody Detected	No HIV Antigen or Antibody Detected
Anti-DNA (DS) AB	Positive	Negative
Rheumatoid Factor	12	<12.5 IU/mL
CCP antibody	<0.5	0-5 U/mL
ANA Screen	Positive	Negative
ANA Titer	1.5120 (H)	<1.40 Negative Titre

**Table 4:** Miscellaneous testing values of the patient. PCR: Polymerase chain reaction.

Test	Patient Values on Admission (Day 1)	Reference Ranges
Chlamydia DNA Genital Swab	Not Detected	Not Detected
Gonococcal DNA Genital Swab	Detected	Not Detected
SARS CoV 2 (COVID 19) Nucleic Acid Amplification PCR Nasopharyngeall Swab	Not Detected	Not Detected
Sickle Cell Prep	Negative	Negative
Parvovirus B19 Quant PCR	<100	<100 Copies/mL
Mononucleosis Spot Test	Negative	Negative

## Discussion

It is essential to consider DGI in addition to lupus flare when treating a patient with a history of SLE presenting with asymmetric joint pain. Lower levels of C3 and C4 in patients puts them at a higher risk of infection due to complement-dependent immune defense against Neisserial infections [4]. Female sex, menstruation, and pregnancy are other well-known risk factors for DGI [5]. Lower complement levels also predispose these patients to more severe infection if left untreated, as in this patient [6]. *N. gonorrhea* can present with various symptoms. An asymptomatic primary mucosal infection is more likely to result in DGI than a primary infection with typical symptoms, which is the case in our patient. Gonorrhea with an asymptomatic presentation may have been acquired days to months before DGI [7]. Asymptomatic primary infection is also more common in women and leads to higher rates of DGI due to delayed diagnosis [8]. DGI has two presentations: arthritis-dermatitis syndrome if there is skin involvement or, less commonly, localized septic arthritis if there is no skin involvement [2]. This patient had a less common presentation with no skin involvement and had symptoms that correspond to the localized septic arthritis presentation of early DGI. The papular skin lesions can start over affected joints and commonly begin as small and erythematous papules if present [8]. 

This patient was diagnosed and treated according to current guidelines for DGI. The diagnosis was confirmed with an Aptima Combo 2 assay endocervical swab that uses targeted amplification to detect Chlamydia trachomatis and *Neisseria gonorrhoeae* rRNA. Aptima was shown in one study to have 91.3% specificity and 99.3% sensitivity for detecting *N. gonorrhoeae*, making it a dependable test for diagnosis [9]. Treatment includes ceftriaxone 1g every 24 hours and doxycycline 100 mg twice daily for seven days. Patients with SLE may need treatment for up to 14 days [3]. Patients are also advised to tell anyone they have had sexual contact with within the last 60 days, so they can obtain testing and treatment [5]. Earlier diagnosis and treatment prevent joint damage and disease burden, including pain and emotional distress, in patients. Early treatment is essential in patients with complement deficiency and illustrates the importance of considering DGI in SLE patients with acute asymmetric mono or polyarthralgia.

## Conclusions

Diagnosing DGI in a patient with pre-existing SLE can be challenging as symptoms of DGI overlap with symptoms of an SLE flare. It is important to consider a diagnosis of DGI in patients with asymmetric polyarthritis, even in the absence of skin or asymptomatic mucosal infection. If patients with DGI are treated early with antibiotics, and the severity of infection is low, full recovery is expected with a low risk of later manifestations.
